# Opportunities and Barriers to Medication Safety in Community-Dwelling Older Adults

**DOI:** 10.1093/geroni/igab046.2332

**Published:** 2021-12-17

**Authors:** Fatoumata Jallow, Elisa Stehling, Zara Sajwani, Kathryn Daniel, Yan Xiao

**Affiliations:** 1 University of Texas at Arlington, Arlington, Texas, United States; 2 University of Tx. at Arlington, Arlington, Texas, United States; 3 The University of Texas at Arlington, The University of Texas at Arlington, Texas, United States

## Abstract

Community-dwelling multi-morbid older adults are a vulnerable population for medication safety-related threats. We interviewed a sample of these older adults recruited from local retirement communities and from primary care practices to learn their perceptions of barriers and enablers for their medication safety. The present study is part of the Partnership in Resilience for Medication Safety (PROMIS) study. One of the aims of this project is to identify barriers and opportunities to improve older adults' medication safety. These interviews were conducted during COVID-19 pandemic conditions. Results from this qualitative study suggest that trust between these older adults and their healthcare providers is an essential component of medication safety. Overarching themes include disruptions in medication management, caregivers caring for each other, patient safety practices or habits, and medication management literacy. Participants also shared strain due to lack of skills to navigate telemedicine visits, trust in Primary Care Providers (PCPs) and pharmacists to prescribe and dispense safely for them, reliance on PCPs and pharmacists to give essential information about medications without having to be asked. Our interviews illustrated large variations in older adults’ perceived role in medication safety, with some developing expertise in understanding how medications work for them and how long-term medications should be periodically reviewed. The types of information needs and supports from PCPs were likely different. Understanding these barriers and enablers for safe medication management can help us develop medication safety improvements for this vulnerable population.

